# Colloidal Lithography for Photovoltaics: An Attractive Route for Light Management

**DOI:** 10.3390/nano11071665

**Published:** 2021-06-24

**Authors:** Rui D. Oliveira, Ana Mouquinho, Pedro Centeno, Miguel Alexandre, Sirazul Haque, Rodrigo Martins, Elvira Fortunato, Hugo Águas, Manuel J. Mendes

**Affiliations:** CENIMAT/I3N, Departamento de Ciência dos Materiais, Faculdade de Ciências e Tecnologia, FCT, Universidade Nova de Lisboa, and CEMOP/UNINOVA, 2829-516 Caparica, Portugal; rdd.oliveira@campus.fct.unl.pt (R.D.O.); p.centeno@campus.fct.unl.pt (P.C.); m.alexandre@campus.fct.unl.pt (M.A.); s.haque@campus.fct.unl.pt (S.H.); rfpm@fct.unl.pt (R.M.); emf@fct.unl.pt (E.F.); hma@fct.unl.pt (H.Á.)

**Keywords:** colloidal lithography, thin-film photovoltaics, photonics, light-trapping, self-cleaning

## Abstract

The pursuit of ever-more efficient, reliable, and affordable solar cells has pushed the development of nano/micro-technological solutions capable of boosting photovoltaic (PV) performance without significantly increasing costs. One of the most relevant solutions is based on light management via photonic wavelength-sized structures, as these enable pronounced efficiency improvements by reducing reflection and by trapping the light inside the devices. Furthermore, optimized microstructured coatings allow self-cleaning functionality via effective water repulsion, which reduces the accumulation of dust and particles that cause shading. Nevertheless, when it comes to market deployment, nano/micro-patterning strategies can only find application in the PV industry if their integration does not require high additional costs or delays in high-throughput solar cell manufacturing. As such, colloidal lithography (CL) is considered the preferential structuring method for PV, as it is an inexpensive and highly scalable soft-patterning technique allowing nanoscopic precision over indefinitely large areas. Tuning specific parameters, such as the size of colloids, shape, monodispersity, and final arrangement, CL enables the production of various templates/masks for different purposes and applications. This review intends to compile several recent high-profile works on this subject and how they can influence the future of solar electricity.

## 1. Introduction

**Figure 1:**
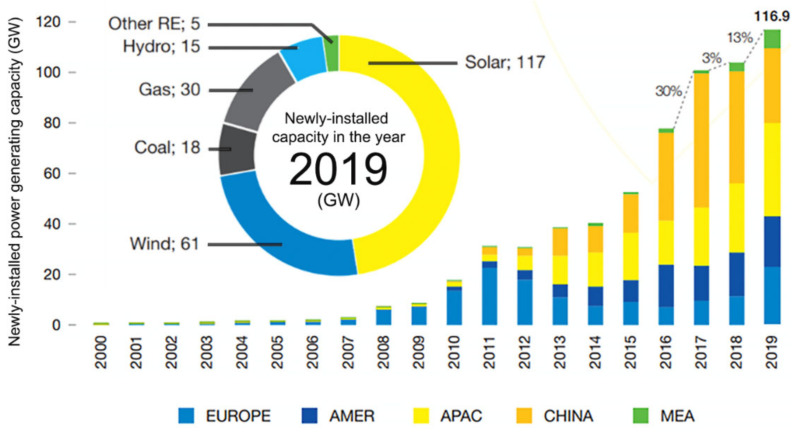
Annual growth of the solar energy market in each development area/country. Although Europe is slowly increasing its production, China is decreasing its yearly rate—overall, the global market is growing largely throughout the years. The inset pie plot presents the net power generating capacity (in GW) added in 2019 for several energy-generating sources, showing that the biggest market growth belongs to solar energy. Adapted with permission from [5]. Copyright 2020 SolarPower Europe.

Highly efficient renewable energy sources and storage devices are needed to deal with the increasingly expensive energetic demands of our society. Considering the depleting fossil fuel stock and the devastating effects of global warming, technologies like photovoltaics (PV) have become one of the leading contenders in this field, as PV offers a broad diversity of devices—each with their potential use and functionality [1,2,3,4]. Recent reports [5,6] show that, despite its current small output, about half of the growth in the electric production capacity worldwide is now held by solar energy systems (Figure 1), with the technology costs decreasing largely. These are indicators of a clear energy transition with large investment, highlighting a tremendous growth potential.

First-generation solar cells based in mono-crystalline silicon wafers convert a large fraction of the incident sunlight energy with an efficiency of up to ~26 %, being still the most commercially used PV technology with a widespread application on rooftops and solar farms. Second-generation solar cells, based on thin-film technologies, have shown signs of becoming a competitive PV class due to potential advantages in low cost, large area, lightweight, solution-process fabrication, and mechanical flexibility [7]. In this category, we can additionally include semitransparent organic and hybrid (organic-inorganic) PV devices, which also tend to be suitable for indoor applications as they work relatively well with diffuse visible light [8,9,10].

Thin-film solar cells enable fast and cheap production methods, such as flexible roll-to-roll processes [9,11]. Moreover, thin devices benefit from lower material usage and thence further cost reduction, also with the potential of increase in the open-circuit voltages, V_OC_ (and consequently efficiencies), due to lower bulk recombination. These are crucial factors at the industrial level for cost-effective production, making the technology attractive for application in affordable solar-powered consumer products such as mobile electronics (e.g., wearable PV), intelligent packaging (e.g., smart labels), electronic devices for Internet of Things (IoT) applications (e.g., smart buildings), and portable medical diagnostic services [12,13,14,15,16,17,18].

The efficiency of the solar cells is inherently limited by the absorber material’s bandgap, as it sets the lower energy limit for absorption [19]. Moreover, thin-film PV suffers from additional absorption losses from the smaller travel path of light within the thin absorber layer [20]. One method to circumvent the first problem would be shifting the incident lower energy photons into higher energy photons [21]. To overcome the second drawback, advanced light management techniques must be used to improve the optical response of the devices. From these, light-trapping (LT) schemes that create challenging conditions for light to escape the device have been the topic of many studies [22,23,24,25,26,27,28,29,30,31]. The development of photonic structures, implemented via nano-patterning methods [32,33], has been fundamental for PV performance enhancements, for instance by extending the absorption onset to the near-infrared (NIR) region of the solar spectrum [34].

As a production technique, microfabrication has been essential to modern science and technology through its role in microelectronics and optoelectronics. Photolithography is the current state-of-the-art patterning technique, and as such, it is the most well-established microfabrication method [35,36]. However, it is primarily limited by its low diffraction-limited resolution, high-cost, and low-throughput. The sizes of the features that it can produce (i.e., patterning resolution) are mostly determined by the wavelength of the radiation used. As such, small features require high-energy radiation and thence complex and expensive facilities and technologies [37,38]. Moreover, photolithography cannot be easily applied to nonpolar surfaces, as it tolerates little variation in the materials that can be used, and it provides almost no control over the chemistry of the patterned surfaces.

With these disadvantages in mind, many alternative techniques have been developed to fabricate nanostructures [39]. For instance, electron beam lithography is capable of fabricating designs with <10 nm resolution, with a high level of acceptance for application in more sophisticated devices where costs are not critical, but is even more limited in terms of patterning speed [40]. Focused ion beam (FIB) lithography has similar advantages and drawbacks, but permits patterning without the use of a resistor or mask [41]. Nevertheless, these techniques do not entirely mitigate the previously mentioned issues of photolithography, such as the cost, low throughput, and the requirement for highly sophisticated equipment. Hence, such disadvantages prompted research for unconventional soft-lithography fabrication techniques [39] as: nanoimprint lithography (NIL) [42,43], hot embossing [44], thermal injection molding [45], light-initiated polymerization (with ultraviolet, UV-NIL, and step-and-flash NIL [43,46]), solvent-based processing [47], and colloidal lithography (CL) [48]. These cost-effective soft techniques have brought focus to the patterning world, as they can be used in nonpolar surfaces—increasing the range of allowed materials, enable large scale patterning, and most importantly, employ industrially attractive fabrication methods due to their ease of use and low manufacturing cost [49].

Among several soft-lithography techniques developed in the last decade, NIL and CL sparked the highest research interest for micro and nanostructuring in photovoltaics [42]. In NIL [50], a pattern is created by pressing a mold into the resist, thus printing the inverse design of the mold. This technique is severely limited when applied to large areas, due to sticking issues from the large contact area between the mold and the imprinted structure, as well as the low pattern fidelity over large areas since the polymer chains in the stamping materials tend to relax elastically. NIL depositions are also strictly reserved for materials that can be molded and cured at moderated temperatures, resulting in a limited range of materials that can be effectively NIL patterned. As such, NIL may not provide the best solution for photonic applications, which usually require dense dielectric or metal oxide materials with a high refractive index for stronger interaction with light [26,51].

The main focus of this study, CL, is presently considered the most promising nano/micro-structuring method for photonic and PV applications [52]. It is an especially interesting technique since it can pattern almost any material, as it is not affected by the aforementioned limitations of NIL. It uses low-temperature steps (<100 °C), therefore not limiting the usage of temperature-sensitive materials (e.g., polymeric-based flexible substrates) or devices (e.g., perovskite solar cells, PSCs) [52], tolerating a wide range of materials and surface chemistries. Properties such as processing simplicity, low cost, and substrate agnostic patterning make CL a highly desirable method [53]. It can also produce well-ordered two-dimensional (2D) and three-dimensional (3D) periodic arrays of nanoparticles from various materials on many substrates. Three-dimensional layers are of tremendous interest for photonic crystal-based applications [49], whereas two-dimensional layers can be used as etching or lithographic masks that can be used for nanofabrication of several structures, specially photonic-enhanced PV devices [29,31,52].

In terms of the CL resolution, it is solely dependent on the colloidal particle sizes that can be deposited, thus allowing nanoscale patterning. However, the smaller the particles the more they become affected by destabilizing Brownian forces which prevent their ordered arrangement in the self-assembly process. Consequently, thus far the minimum feasible resolution of CL ranges between 50 to 200 nm [22,54], which is comparable to that of state-of-the-art hard-lithography (more costly) systems, such as photolithography (set by the diffraction limit of UV light), but not as low as the resolution of E-beam or FIB (order of nanometers). Nevertheless, research means are underway to further improve the CL resolution, for instance by operating at low temperature to hinder the Brownian diffusion [55].

This article provides an overview of the present panorama of CL, exploring its working concept and the patterning materials, with a focus on its last-generation PV-related applications.

## 2. Colloidal Lithography (CL) Methodologies

**Figure 2:**
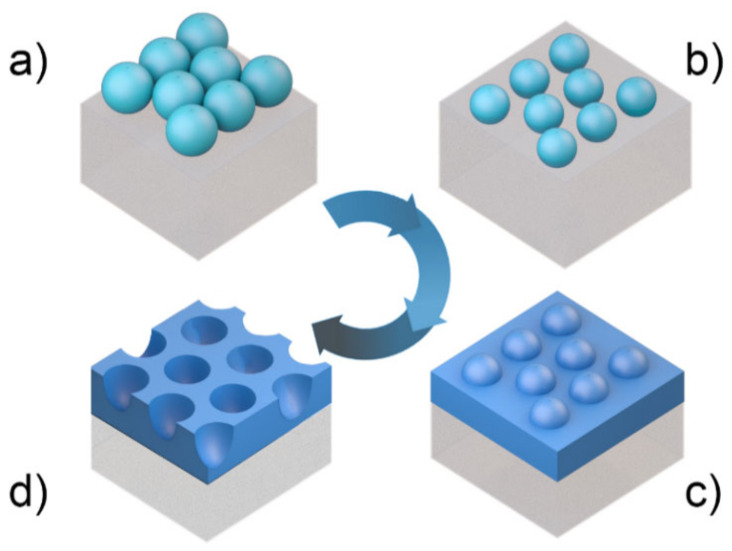
Illustration of the colloidal lithography (CL) main steps, depicting the sequence of (**a**) the deposition of colloidal particles on a surface, (**b**) reactive ion etching (RIE) for particle shaping, (**c**) material deposition, and (**d**) lift-off of the colloids leaving only the patterned material on the surface.

The use of colloids in lithography has been studied for about 35 years, with the continuous development of nano and microfabrication methods reaching increasing potentialities [39]. The methodology (Figure 2) generally comprises two main stages: the patterning mask preparation (Figure 2a,b), followed by the nano/micro-structure production (Figure 2c,d). The process starts with a colloidal deposition technique which is further described in Section 2.2, where we present a large set of procedures that use self-assembled colloidal arrays for surface patterning. The variety of methods that can be used for the colloidal array formation, as well as for the subsequent structure production, shows the high versatility of this method for implementation in various applications.

### 2.1. Colloidal Self-Assembly

Colloid particles are an important class of materials, sharing properties with bulk and molecularly dispersed systems. Their behavior is mostly governed by the particles’ size, shape, surface area, and surface charge density [56]. Several techniques and protocols have been developed to synthesize highly monodispersed colloidal spheres with diameters ranging from a few to thousands of nanometers.

For CL applications, any particle material can potentially be used to create the self-assembled colloidal mask in the first 2 steps of Figure 2. However, the preference lies in colloidal materials that: (1) can be synthesized with precise monodispersed particle sizes; (2) allow highly-selective etching (RIE) in step b) of Figure 2; and (3) can be easily removed by chemical lift-off in step d) [54,57,58,59,60,61,62,63,64]. In view of that, the most synthesized particle materials for use in CL have been polystyrene (PS), polymethyl methacrylate (PMMA), and silica [65]. Good phase stability, together with a narrow colloidal size distribution (less than 5% for the typically employed microspheres), has been achieved by suspension [40], emulsion [66], and dispersion polymerization [64,67] synthesis techniques.

**Figure 3:**
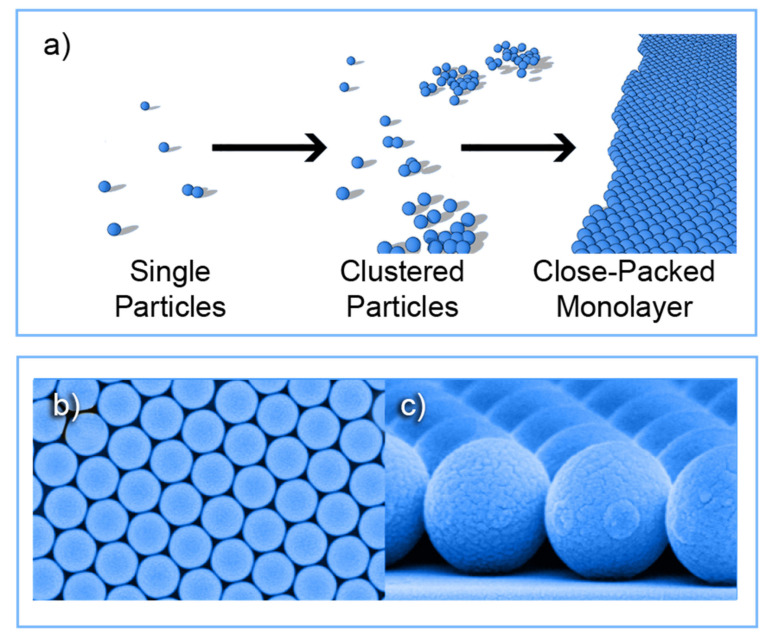
(**a**) Illustration of the self-assembly of colloidal particle structures forming a hexagonal close-packed array (also known as honeycomb), which results in the highest in-plane packing density; (**b**) Top-view and (**c**) cross-sectional scanning electron microscopy (SEM) images of honeycomb arrays of 800 nm PS spheres. Adapted with permission from [69]. Copyright 2021 Elsevier.

The self-assembly of colloidal particles, crucial for next-generation surface and volume nanostructuring applications, consists of their spontaneous arrangement into ordered superstructures (Figure 3) [68].

Based on the type of dominant force driving the self-assembly, these methods can be organized into four classes: physical (process dominated by shear forces, adhesion, and surface structuring), fluidic (by capillary forces, evaporation, surface tension), external fields (by electric and magnetic fields) and chemical (by chemical interaction, changing the surface charge or creating binding sites) [70].

In 1981, a lithographic method using self-assembled PS monolayers as a mask was first proposed by Fisher and Zingsheim [71]. Afterwards, Deckman and co-workers successfully increased the mask area for patterning [72]. Owing to the size, shape, and monodispersity, colloidal particles can self-assemble into 2D or 3D extended periodic arrays, but the 2D colloidal crystals are those that captured the most attention for PV application [53,73,74].

The production of self-assembled arrays of colloidal particles is the starting point of the CL process, which utilizes the close packing of such colloidal crystals to fabricate long-range ordered nano/micro-structures in/with any material [68,70,73].

Interesting examples are the fabrication of nanoporous templates [75], 3D photonic bandgap structures [76], and thin-film nanocrystal solids for electronic devices [77]. For instance, it has been shown that spherical colloidal particles coated with liquid crystals, or other materials having nematic degrees of freedom, can form composite materials that exhibit point defects with sp and sp^3^ valences. For future applications, the most intriguing aspects of colloidal particles are their potential utility as building blocks, capable of mimicking molecular self-assembly through covalent and non-covalent interactions, to created artificially-designed materials [78].

### 2.2. Deposition of Colloidal Arrays

**Figure 4:**
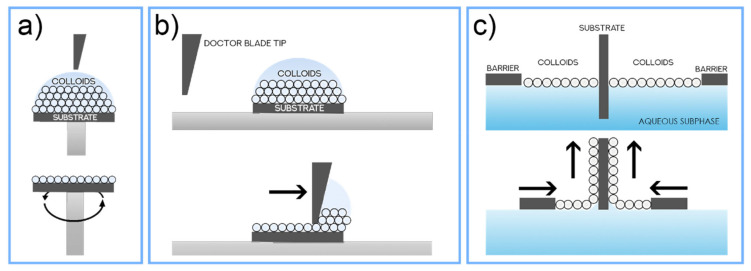
Production of a colloidal monolayer using (**a**) a spin-coating technique, (**b**) doctor blade trough, (**c**), and a Langmuir–Blodgett trough.

Three techniques should be emphasized when considering the initial step of colloidal monolayer deposition on the substrate of the CL method (see Figure 2a). These are spin-coating, doctor blade, and Langmuir–Blodgett sketched in Figure 4 and described in this sub-section.

The spin-coating technique (Figure 4a) can be considered a simple process for rapidly depositing thin coatings onto relatively flat substrates [54,79,80,81]. A spinning fixture holds the substrate (often using vacuum to clamp and position the substrate in place), and the coating solution/dispersion is then dispensed onto the surface. The revolving action causes the solution to spread out and leave behind a uniform coating of the chosen material on the surface. Due to its short time of production, combined with its simplicity and low cost, this method is useful in industrial conditions as far as small-area batch coating processes are concerned. However, it is not compatible with large-area deposition, and the resulting colloidal films tend to be less uniform than those produced by dip-coating methods such as Langmuir-Blodgett [82].

Spin-coating experiments have mainly been designed to deposit small-area nanosphere monolayers, severely limiting the application of these films as physical masks. Therefore, important research parameters have been optimized to prepare high-ordered colloidal films with different diameter nanospheres on larger scales. For instance, by adjusting the spin speed and acceleration, Chen et al., (2013) [54] spin-coated long-range ordered colloidal crystal films of PS spheres with diameters of 223 nm, 347 nm, 509 nm, and 1300 nm. Furthermore, for the 509 nm of spheres’ diameter, the team also used these conditions to inspect the relation between the monolayer coverage area and spin parameters. It was found that with the increase of the spin speed and acceleration, the monolayer coverage areas oscillated, with the largest ordered areas (near 100% of 25 mm × 25 mm × 0.5 mm quadrate and 3-inch circular silicon substrates) being achieved at a speed of 1700 rpm and acceleration of 600 rpm/s. Chen’s results thus revealed the successful preparation of monolayer and bilayer films of PS nanospheres with four different diameters. In the only structure that was considered to be with reasonable hexagonal close-packed ordering, both monolayers and bilayers could be found, which is not suitable for colloidal lithography applications.

Another impactful development was reported by Park et al. [81], who introduced polyoxyethylene (12) tridecyl ether (PEO-TDE) as a surfactant for the spin-coating of PS nanosphere monolayers onto Si wafers and glass substrates, under ambient laboratory conditions, with optimal surfactant properties, as opposed to the conventional highly toxic Triton X-100 surfactant. Low viscosity and surface tension cause this mixture to show excellent wettability, which results in superior coverage and uniformity [81].

Another simple, but highly scalable process for nano/microspheres deposition is the doctor blade coating or blade coating technique (Figure 4b). This method is widely used in the textile, paper, photographic film, printing, and ceramic industries to create highly uniform flat films over large areas [83]. An immobilized blade (or rod) applies a unidirectional shear force to a slurry that passes through a small gap between the blade and the substrate. This is a roll-to-roll compatible method that has played a crucial role in ceramic processing to produce thin, flat ceramic tapes for dielectrics, fuel cells, batteries, and functionally graded materials. A simplified doctor blade coating process was developed by Velev et al. [84], based on an evaporative colloidal assembly technology that relies on capillary forces to drive and merge colloidal particles into crystalline structures with thicknesses ranging from a single monolayer to a few layers. Inspired by this technology, Yang et al., (2010) [85] reported a roll-to-roll compatible doctor blade technology for producing highly ordered colloidal crystals (mainly polymer nanocomposites) and macroporous polymer membranes. The resulting 3D-ordered structures exhibited uniform diffractive colors, and Yang has shown that the templated macroporous membranes with interconnected voids and uniform interconnecting nanopores can be directly used as filtration membranes to achieve the size-exclusive separation of particles.

Lastly, the Langmuir–Blodgett (LB) method [86] (Figure 4c) consists of the compression of nanoparticles, floating in an air-liquid interface, into monolayers—Langmuir films—and its transferal onto immersed solid substrates via vertical dipping [87]. This technique offers the possibility to obtain highly ordered, well-defined, controlled mono/multilayers, ultimately serving the patterning purposes for CL applications [88]. Common LB-deposited materials have amphiphilic molecules with two distinct regions: a hydrophilic head group (water affiliation) and a hydrophobic tail group (water repulsion). They must be soluble in organic nonpolar and water-immiscible solvents (ethanol, diacetone, chloroform, benzene, among others [89,90]), with water-insoluble amphiphilic molecules forming a floating monolayer at the air-water interface. Long-chain fatty acid and lipid molecules are examples of typical LB-deposited materials, but the method has also been found successful (highly precise) for the patterning of close-packed monolayers of colloidal spheres, as for CL.

The two main steps of the LB technique are the preparation of a floating self-assembled colloidal monolayer at the air-water interface (Langmuir-film) and its deposition on a solid substrate [91]. At first, the colloidal particles are dispersed in a volatile and preferably water-insoluble solvent to prepare the colloidal dispersion. Then, small amounts of this solution are carefully deposited and spread onto the air-water interface at the LB trough. Afterward, the volatile solvent evaporates, and the LB barriers are compressed accordingly to force the formation of a self-assembled close-packed colloid monolayer at the interface [88]. Finally, the immersed substrate is withdrawn vertically from the aqueous subphase, while the lateral barriers continue to close in towards the substrate, at controlled rates, therefore transferring the colloids stabilized at the air/water interface to the upwards moving substrate [86], resulting in a successfully deposited monolayer colloidal film. Multilayer films can also be engineered by successively subjecting the previously deposited substrate to further cycles of LB deposition. These multilayers have been considered model membranes due to their remarkable 3D uniformity; and offer potential application as photonic waveguides and in breakthrough optoelectronic, nonlinear optical, and sensory devices [92,93].

Surface and interface chemistry is of paramount importance for defining how the colloidal particles float and are packed [32,33,81,94]. For instance, relatively small PS colloids with sizes close to visible wavelengths (under 1 µm) tend to sink into the aqueous subphase, contrasting with larger ones that typically float [94]. The fabrication of monolayers by interface coating methods as LB has been subject to numerous studies, varying the size of colloids, the amount of solution, temperature, deposition angle, and others [22,95,96,97].

It is also believed that the use of surfactants in the aqueous subphase may enhance the floating and Langmuir-film production of colloidal particles at the air/water interface, with larger areas and mechanical strength, similarly to the solutions presented by Vogel et al., (2011) [94] for the spin-coating method. Surfactant molecules tend to occupy the media interfaces and join the incoming colloidal particles together, thus opposing their dispersion caused by the Brownian motion. The surface assembly forces tend to enlarge the array area, increasing the monolayer order and coverage [95]. There is also a reduction of surface tension at the interface due to the presence of the surfactant, which favors colloidal particle movement along the interface to find their lowest energy configuration, resulting in an optimally-ordered hexagonal close-packed monolayer [98]. Adding the surfactant, however, may introduce undesired contamination to the interface. Therefore, care must be taken to avoid the transfer of substantial amounts of contaminants to the substrate during the LB lift-up process, to avoid imperfections in the deposited colloidal array [79].

From the three methods mentioned above, the Langmuir-Blodgett method combines quality, versatility, and scalability, being the headmost characteristics for the fabrication of high-quality 2D or 3D crystalline films. Furthermore, the capacity to precisely control the deposition of each layer in a layer-by-layer process, the ability to choose different particle sizes for each deposited layer, and the possibility for this method to be adapted to fast industrial production techniques such as roll-to-roll processing [22,99,100,101] make it an outstanding candidate for the first step of the CL process. These advantages are demonstrated in the research of O. Sanchez-Sobrado et al. [29,33,48] that has revealed outstanding results of thin-film solar cells enhanced with photonic front structures that were patterned via CL using highly-uniform LB-deposited colloidal masks.

### 2.3. Colloidal Masking for Surface Patterning

After achieving a good monolayer of close-packed colloidal particles, as previously described, it is then necessary to define how one can effectively use such array as a mask to achieve the desired microstructures in the targeted material (recall Figure 2). The final structures and properties achieved by the CL process are highly dependent on the prepared mask of material and packing [102,103]. Although attaching functional molecules or coating materials to colloids offers various possibilities for additional tuning of their properties [104], most polymeric or silica colloids end up being straightforwardly assembled into monolayers without functionalization, for further processing in CL.

The use of the originally deposited close-packed colloids (Figure 2a) as a mask allows only a limited exposure area in the interstitial spaces of the hexagonal array. Therefore, increasing the inter-particle distance in non-close-packed arrays (Figure 2b) is an important tool to optimize the masked area on the surface, at the expense of requiring an extra intermediate step of physical etching of the colloids.

Still, using the simpler CL version with close-packed 2D colloidal crystals as etching masks, triangular nanoparticles [105], nanodots [106], and thin-films with nanohole arrays [107], nanotips [108], or nanopillars [109] have been fabricated on several substrates (such as polymer-based, silica, and silicon) [65,69,97].

**Figure 5:**
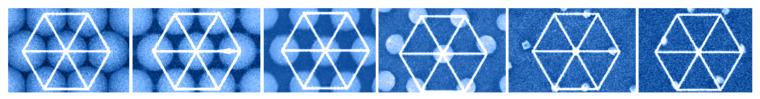
Schematic illustration of the reduction of the colloidal spheres dimension (with 0.5 μm initial diameter) and increase of their spacing in the array, via low-frequency plasma etching, with increasing etching time (left to right). Adapted with permission from [60]. Copyright 2021 MDPI.

Regarding the formation of non-close-packed monolayers (Figure 2b), dry etching methods have been used (such as reactive ion etching, RIE) to reduce the size of the spheres after their deposition, and therefore increase their inter-space distancing in the array (Figure 5).

A recent study from Yun Chen et al., (2019) [60] has shown that low-frequency plasma etching (40 kHz) can be used to produce PS nanospheres-based arrays with smooth surfaces, doubling the etching rate when compared to high-frequency systems. This study revealed that low-frequency RIE processes are dominated by a thermal evaporation etching mechanism, different from the atom-scale dissociation mechanism that underlines the high-frequency etching. It was found that the PS features size can be precisely controlled by adjusting the etching time and/or power. By introducing oxygen as the assisting gas in the low-frequency RIE system, one can achieve a coalesced PS particle array and use it, for instance, as a template for metal-assisted chemical etching which can significantly improve the aspect ratio of silicon nanowires to over 200 due to their improved flexure rigidity.

**Figure 6:**
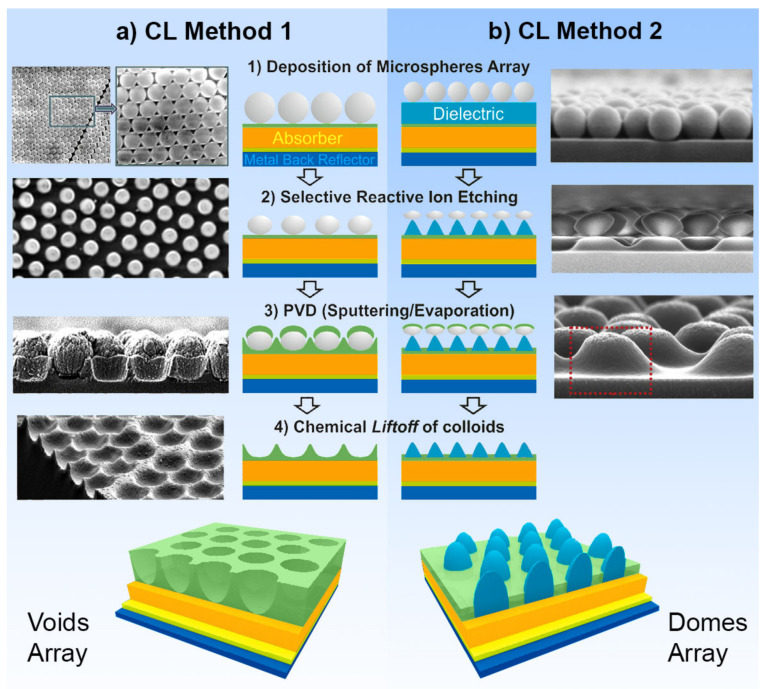
Depiction of two different CL methods used to create distinct geometries of photonic microstructures for light-trapping, integrated in the front contact of thin-film solar cells, arranged in non-closed-packed hexagonal (honeycomb) arrays of semi-spheroidal voids (**a**) or domes (**b**) Adapted with permission from [52]. Copyright 2021 Elsevier.

RIE has also been used in the CL fabrication of optimized photonic front structures for light-trapping in thin-film solar cells, which is crucial for increasing light absorbance in the absorber layer and subsequently the performance of the devices. Recently, two efficient four-step approaches were described by Mendes et al., (2020) [52] that can produce two types of geometries based on arrays of semi-spheroidal voids or domes, as shown in Figure 6. Briefly, for both cases, this method starts with the deposition of a monolayer of close-packed colloidal PS microspheres (LB method), followed by RIE producing a non-close-packed array.

The main difference between both approaches occurs in the first two steps. In the first method (Figure 6a), the selective RIE only acts on the particles, so the final void-like structures are defined by the subsequent deposition of material in the inter-spaces between particles (as in Figure 2c,d). Although in the second method (Figure 6b), the less-selective RIE process also etches the underlying layer, ultimately defining the final dome/cone-like structures obtained.

Using etched nanospheres as molds/masks in processes such as metal deposition, infiltration, or imprint, it has been possible to produce ordered arrays of spherical voids [110] and nanoshells [111,112]. Through dewetting around nanospheres, nanorings of polymers [113], carbon nanotubes, or nanoparticles [63] can be obtained. Resorting to site-selective deposition or etching, nanospheres with asymmetric shapes or functional features have been produced [114], difficult or impossible to obtain by other synthetic routes.

After the deposition of the intended material onto the RIE-shaped colloidal mask in step c) of Figure 2, a lift-off treatment removes the colloids (step d) leaving only the microstructured material on the front surface. After this process, undesirable colloidal residues may be found in the areas previously occupied by the particles, due to incomplete removal. In such cases, besides the chemical removal (e.g., with toluene), both oxygen plasma [115] and thermal annealing [116] treatments can also be used to remove the polymeric particle residues which are usually quite volatile at temperatures around 100 °C.

## 3. Photonic Crystals

**Figure 7:**
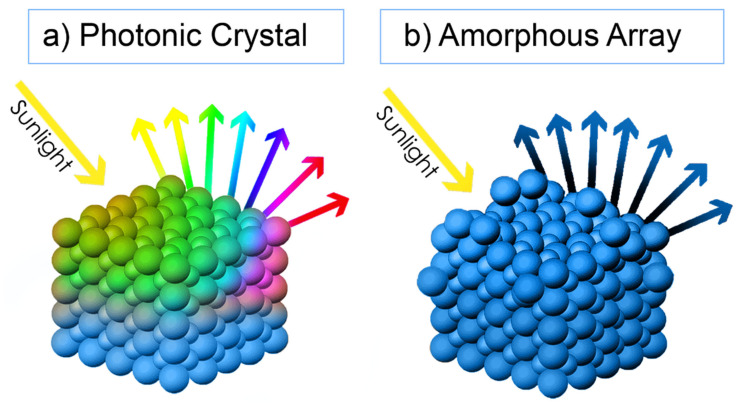
Schematic optical interaction of (**a**) an ordered colloidal crystal and (**b**) an amorphous colloidal array under white light. The structural color from the colloidal crystal changes depending on the viewing angle, while that of the amorphous array remains nearly unchanged.

The previous section showed that there is a wide range of nano/micro-structure designs that can be engineered with CL techniques for various technologies, whose dimensions are chiefly set by the size of the masking particles. The colloidal particles’ size propinquity to visible light’s wavelengths will therefore grant the fabricated structures excellent interaction properties with this type of radiation (Figure 7) [57,70,117,118,119].

It is important to note that photonic crystals have structural similarities with common crystals but have no direct relationship with crystalline materials. The dimensionality of the photonic crystal is defined by the length(s) in which the dielectric constant varies periodically, and they can be represented by basic 1D, 2D, and 3D crystals. However, only 3D photonic crystals allow for omnidirectional photonic bandgaps [120] that are an optical analog of the energy bandgap of the crystalline network.

**Figure 8:**
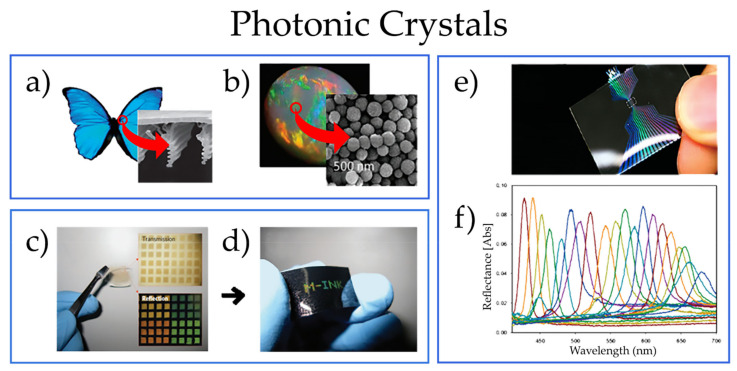
Natural photonic crystals (left): (**a**) photo showing the blue iridescence and SEM image of the 1D structure of the Morpho butterfly, (**b**) photo of an opal gemstone, and SEM image of the silica sphere structure within it. Adapted with permission from [121]. Copyright 2021 The Royal Society of Chemistry. Artificial fabricated photonic crystals deposited on flexible substrates made of semitransparent: (**c**) photonic crystal films and (**d**) photonic crystal films with anti-transmission black tape as the transferred substrate, which blocks backlight transmission [122], as well as (**e**) photo and (**f**) reflectance spectra of stripe-patterned composite photonic crystals with 20 different optical bandgaps. Adapted with permission from [123]. Copyright 2021 John Wiley and Sons.

In nature, one can find many examples of natural photonic crystals (Figure 8), such as in wings of butterflies and natural opals (Figure 8a,b). These natural crystals are composed of periodic microstructures whose scattering and transmission properties strongly depend on the incident light frequency, thus displaying brilliant colors, which have inspired artificial designs (Figure 8c,f).

Due to their unique characteristics, photonic crystals fabricated via colloidal assembly have attracted much interest for various prospective applications, ranging from gas sensing to optical filters [124,125], photonic papers [126], inkless printing [127], flat reflective displays [128], optical devices, photochemistry, and biological sensors [70]. Recent developments have further enhanced their complexity using non-spherical particles [129,130], binary colloidal dispersions [131], as well as controlled production of 3D defects (acting as optical cavities) within the crystals [132,133]. Several approaches have been developed enabling defect engineering controlled to a great extent, such as surface micromachining which allows for symmetries other than face-centered cubic [117,134].

Self-assembly has a crucial role in the fabrication of photonic crystals with a photonic bandgap in the visible and near-infrared region [135]. For instance, freestanding films have been fabricated by the layer-by-layer assembly [136], solution casting [137], surfactant-assisted deposition [138], and filtration of dispersions of materials using membrane filters [61]. A facile approach to fabricate such large asymmetric free-standing 2D array films is by forming 2D colloidal particle arrays at the air−water interface, as in the LB method [139]. Nevertheless, besides LB, colloidal photonic crystal growth via self-assembly of monodispersed colloids can involve, as previously mentioned, various fabrication methods such as controlled evaporation, spin coating, shear growth, among others.

Although the fabrication of 1D or 2D photonic crystals is relatively straightforward, adapting the conventional patterning techniques to fabricate 3D crystals remains a challenge. This originates from the stringent constitutional quality, and functional requirements. Several methods have been proposed in this respect. The most economical and direct approach to fabricating 3D photonic crystals is also by the self-organization of colloidal particles. The most inexpensive and direct approach to fabricating 3D photonic crystals is also by the self-organization of colloids that can be used as a template. Inverse opals materials with a high degree of periodicity in three dimensions are important templates for the design of photonic crystals. One method used to prepare these photonic crystals consists in infiltrating the void spaces between spheres in a colloidal crystal template with the desired material in solution phase (sol-gel), which is subsequently solidified. The subsequent removal of the templating spheres leaves a structured photonic crystal [140]. The sequential passivation reactive ion etching (SPRIE) method, [117] as the name suggests, relies on sequential passivation and reactive ion etching reactions using C_4_F_8_ and SF_6_ plasma chemistries. It allows the addition of the third dimension using a simple and robust protocol for direct structuring of silicon-based 3D photonic crystals. Through a single processing step, SPRIE transcribes 2D colloidal crystal arrangements into well-ordered 3D architectures. The lateral etch extent controls various 3D topologies, useful in the delamination of 3D photonic crystals slabs or for the insertion of structural defects [117].

Alternatively, direct writing or single-step processing techniques have emerged as powerful tools for rapid and scalable 3D photonic crystals fabrication. Multiphoton polymerization lithography seems an attractive scenario, as it allows for unprecedented control of the crystal geometry and the defect incorporation, although it suffers from low throughput due to the serial writing procedure [117]. By infiltrating the interstices of polymer latex colloidal crystals with inorganic materials, and subsequently burning out the polymer latex, ordered macroporous films known as ‘inverse opals’ can be prepared. Inverse opals can have higher reflectivity over wider optical stop gaps (which prohibits light from propagating in only some directions) [121] due to the higher refractive index mismatch between the spheres and the medium. Such structures can also have 3D photonic bandgaps in the very high-frequency regions. Despite their bandgaps being very narrow, these can be reduced even further with the introduction of small defects, since they exhibit wider stop gaps and broader mechanical stability. For the production of inverse opal structures, colloidal templates of inorganic colloids (silica) can be used. Within this process, composites with polymeric materials are formed and then converted to polymeric inverse opal structures via the removal of the inorganic particles using selective etchants such as hydrofluoric acid [141].

Non-crystalline colloidal arrays—photonic glasses (see Figure 8b)—have also much interest for certain applications. These structures consist of aggregates of monodisperse colloids with short-range order, over a range of a few particles, that can be detected from the structure’s diffraction pattern. Although photonic crystals can be used to manipulate ballistic photons, photonic glasses are useful in controlling light diffusion. The random structures of designed uniform colloids can interact strongly with light and produce unusual diffusion phenomena, including random lasing, angle-independent color, and light localization. These disordered monodisperse and short-range ordered particle structures have been produced by destabilizing the colloids with the control of the salt concentration, or by adding particles of different sizes to the colloidal solution [141].

## 4. Photovoltaics Enhanced with Micro-Structuring

The amazing light-interaction properties of wavelength-sized structures overviewed in the previous section have motivated their development for optical manipulation in PV devices, aiming for maximum sunlight conversion to electrical power. In particular, thin-film solar cells suffer from significant absorption losses, relative to thicker wafer-based cells, due to their diminished absorber thickness. As such, advanced light management techniques are necessary to compensate for such losses and ensure that high efficiencies are achieved [20,33]. As was already introduced, nanophotonic elements in the wave-optics regime are seen as a promising method to efficiently trap light inside the thin absorber material, thus boosting its broadband absorption, as further discussed in Section 4.1 [142].

The self-assembled templates from colloidal spheres can provide monolayers with long-range order throughout large device areas, thus providing an inexpensive and easily scalable mask to engineer materials with the physical parameters appropriate for efficient light-trapping (LT) in solar cells. This has led to a significant interest in CL methods for photovoltaic devices, which is the focus of this review. Nevertheless, the CL applications in the PV field are not limited to the integration of LT structures. In the next sub-sections, we will highlight two other promising nano/micro-structuring solutions that have also been demonstrated with CL: namely for transparent electrodes (Section 4.2) and self-cleaning functionality (Section 4.3).

A review article by Wang (2018) [73] described that CL can produce several different patterns and geometries that could form an anti-reflective LT mechanism. It may include Janus particles, hexagonal and non-close-packed single layers, double layers, free-standing films, and template-induced arrangements. The nanostructures obtained by this process can already be promptly applied to many different areas. Furthermore, this technique can also be adapted by tweaking the experimental parameters, such as the dimension of the spheres, the morphology of the surface, and chemical composition, thereby increasing the spectrum of possible applications.

Micro-meshed electrodes (MMEs) obtained by CL have been one of the most promising approaches to produce industrial-compatible transparent conducting materials (TCMs), with excellent optical transmittance and electrical conductance, composed of TCO/metal/TCO multilayers (TCO = transparent conductive oxide). In particular, replacing the intra-layer metallic film (usually silver) with a micro-grid of the same material has allowed additional degrees of freedom to optimize the TCM performance, namely enabling much higher transparency in the red-NIR (near-infrared) spectral range while maintaining highly conductive TCMs [62].

Besides efficiency, the outdoor reliability of PV systems is another crucial factor necessary for their widespread deployment. Solar panels tend to lose efficiency with time mainly due to unavoidable environmental degradation. Phenomena such as the formation of hot-spots (areas of large heat dissipation) caused by partial shading of solar cells (e.g., due to debris/dirt deposits) can be responsible for pronounced efficiency losses that, for instance, have reached 11% in three days and 65% after six months in certain power plants [74,143]. Therefore, decreasing unwanted processes that block the amount of sunlight reaching the cell, such as dust and other accumulation of particles, merits particular attention due to their inevitability and ubiquity [74].

Most current solutions to this include the mechanical cleaning of the devices [31] to expel dust specks via four types of techniques: the robotic method, air-blowing method, water-blowing method, and ultrasonic vibration method. Nevertheless, such type of active mechanical methods requires a power source for enabling the self-cleaning mechanism. Moreover, manual cleaning can also create cracks on the PV panel surface due to harsh brushing which will further deteriorate PV performance. Moreover, very small particles cannot be removed effectively by a manual cleaning process. On the other hand, the use of a self-cleaning coating, as no PV panel movement is required for its working function, is way simpler and more fitting to PV applications [74]. Another active method—the electrostatic cleaning method [144]—expels the surface dust through electrostatic standing and traveling waves, due to an existing electric curtain. The electric curtain consists of a series of parallel electrodes embedded in a dielectric surface, across which are transmitted oscillations in the electrode potentials. During this process, the standing wave oscillates the dust particles up and downward while a traveling wave does the same process in a horizontal direction.

Concerning passive coating methods, they employ either a superhydrophilic or superhydrophobic film on the outer PV surface. Superhydrophilic coatings reduce the amount of dirt through photocatalytic reactions, while superhydrophobic coatings potentiate the formation of water droplets and their roll-off, carrying away the dirt from the surfaces with minimum water usage [31,74]. To allow water (or other liquids) droplets to effectively roll down a surface, different superhydrophobic-oriented strategies are being investigated mainly via surface micro-structuring, mimicking natural processes such as the skin of certain plant leaves with self-cleaning capability [145], as further detailed in Section 4.3.

**Figure 9:**
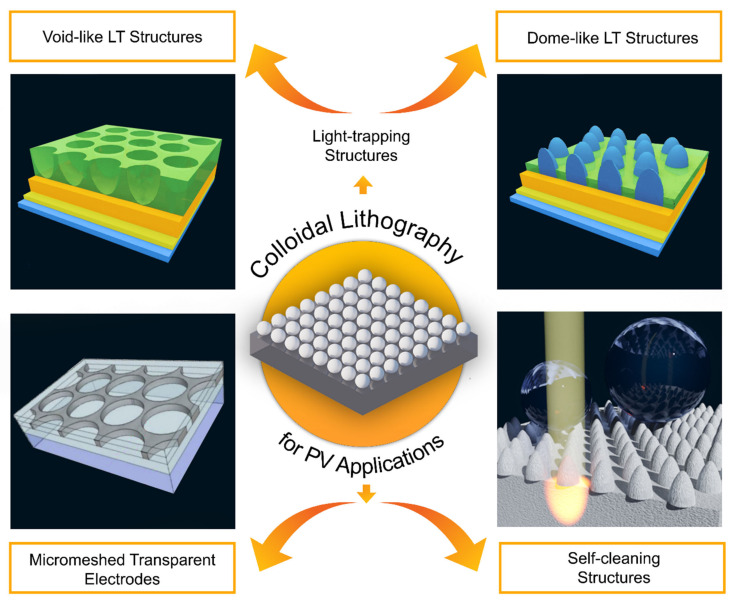
Illustration of the distinct applications of CL for micro-structuring in photovoltaics. Adapted with permission from [62]. Copyright 2021 Elsevier. Adapted with permission from [52]. Copyright 2021 Elsevier. Adapted with permission from [31]. Copyright 2021 John Wiley and Sons.

The aforementioned applications reveal that CL offers a wide range of promising possibilities for the advancement of PV-related technologies, as illustrated in Figure 9 and elaborated in the following sections.

### 4.1. Light-Trapping in Photovoltaics

Light-trapping (LT) structures are a critical enabling factor in PV technology, as they improve the absorption of incident photons, therefore impacting its conversion efficiency [23,29,31,51,52,62,146,147].

On one hand, reflection losses are an unavoidable shortcoming in all types of PV technologies. Proper index matching in the surface—using materials with an index between that of the absorber material and the light incidence medium—can help mitigate this problem. Photonic structures can further diminish these losses in a broad wavelength range—by providing geometric index matching.

On the other hand, these structures also bring about LT mechanisms to help with light management within the device. As previously mentioned, this is particularly important for thin-film solar cells, where the short optical path is not enough to absorb all the incoming solar radiation, and it can be useful also for other emerging solar technologies [148]. This shortcoming has also been severely hindering flexible thin-film PV technology from achieving its market potential. As a matter of fact, many thin solar cells have so far only reached modest efficiencies (~14%) compared to those of conventional cells based on rigid silicon wafers (22–25%). Therefore, there is much improvement potential in thin-film PV with the implementation of effective LT techniques, mainly as a means to make the cells optically thicker but without increasing their physical thickness (to allow efficient charges collection) [52].

Here, a detailed analysis is presented of different types of photonic structures integrated via CL methods for LT in thin-film solar cells, particularly based in silicon and perovskite PV materials, enabling the development of high-efficiency flexible devices.

#### 4.1.1. Computational Design and Optimization of Photonic Solutions

The resonant nature of wavelength-sized photonic structures substantially limits the parameter space in which their optical effects can provide exceptional absorption improvements across the relevant sunlight spectrum. Specifically, the wave-optical front features (as those integrated via the CL methods of Figure 6) need to provide a gradually varying effective refractive index, from the air towards the absorber layer to minimize reflection. Simultaneously, their geometry must interact with the incoming light to produce strong scattered fields preferentially directed into the higher index absorber layer. Therefore, before experimental implementation, it is crucial to perform a rigorous screening based on modeling, to understand the influence that the parameters of the LT structures have on such effects, and then appropriately search for the best parameter set that allows the highest photocurrent enhancement in the devices [142,149,150].

**Figure 10:**
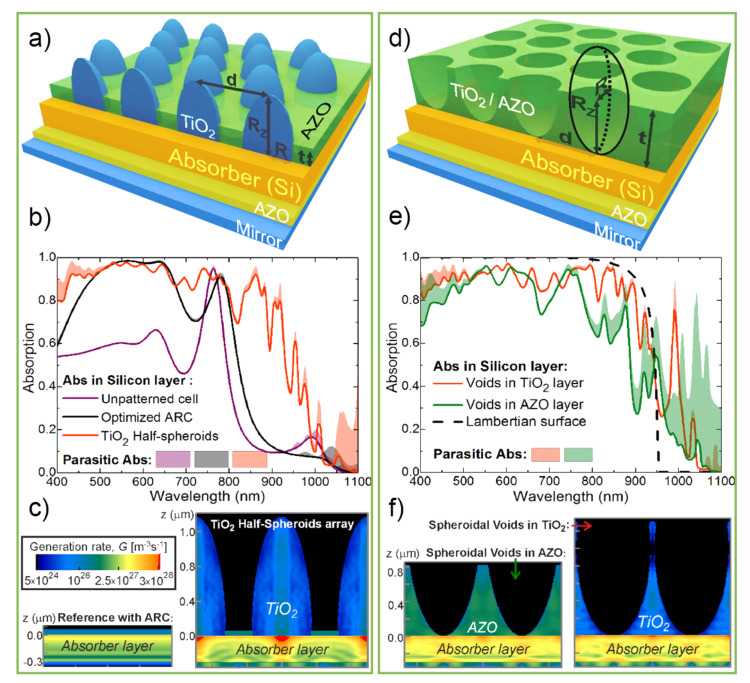
Electromagnetic modeling results of two types of LT geometries (sketched in **a**,**d**) composed of hexagonal arrays of TiO_2_ half-spheroids (**a**–**c**) or semi-spheroidal voids in a TiO_2_ or AZO layer (**d**–**f**), both integrated into thin-film (300 nm) Si solar cells. The results show the light absorption, Abs, spectra (**b**,**e**) and photogeneration rate, G, profiles (**c**,**f**) of the optimized photonic front structures, compared with flat reference cell structures without LT or with a standard AZO antireflection coating (ARC). The G profiles reveal much higher carrier generation in the cells with the photonic elements due to the enhanced broadband absorption. Adapted with permission from [142]. Copyright 2021 Elsevier.

Theoretical studies reported by Mendes et al., (2016 and 2018) [142,149] have sought for designs of wavelength-sized structures optimized towards maximum broadband light absorption, with the double aim of enabling the reduction of the absorbing layer thickness (potentiating flexibility) while improving the efficiency of thin solar cells (Figure 10). Two geometries of front-located photonic structures were pointed out, compatible with the CL fabrication methods of Figure 6, and computationally optimized to maximize absorption without degrading the electrical performance of the devices (by avoiding increased recombination since the absorber layer remains flat, i.e., it is not corrugated as occurs with texturing).

These LT structures are composed of honeycomb arrays of semi-spheroidal features either made of domes (TiO_2_ half-spheroids separated by a flat aluminum-doped zinc oxide—AZO—layer—Figure 10a) or voids (semi-spheroidal holes in a layer of either TiO_2_ or AZO—Figure 10d). The electromagnetic field distribution simulations in the thin-film solar cell structures were carried out using a 3D Finite Difference Time Domain (FDTD) method.

The modeling results show that the structures patterned on the front of the cells drastically reduce reflection losses at short wavelengths—Figure 10b,e (at energies above the absorber bandgap)—via geometrical refractive index matching with the cell media. They also boost the absorption of longer wavelengths by increasing their path length via light bending and coupling with wave-guided modes confined in the absorber layer.

These combined effects (antireflection and light scattering) lead to a substantial broadband absorption enhancement in the absorber material, which allows reducing its thickness without lowering the output current.

When evaluating the different LT geometries, it was found that the optimized absorption spectra attained with the two types of structures do not differ significantly. Nevertheless, the highest photocurrent gains were generally provided by the void geometry due to higher NIR absorption enhancement, as it allows a higher degree of angular spreading of the scattered light. Simultaneously, the domes tend to act as micro-lenses that instead focus the scattered light in localized hot-spots located beneath them, as observed in the optical generation profiles of Figure 10c,f.

Pronounced photocurrent enhancements, up to 37%, 27%, and 48%, are demonstrated with honeycomb arrays of semi-spheroidal dome or void-like elements front-patterned on the cells with ultrathin (100 and 300 nm thick) amorphous, and thin (1.5 μm) crystalline silicon absorbers, respectively [142].

The geometrical optics limits for photocurrent enhancement via LT are known as the Lambertian or Green broadband absorption limits. Isabella et al. [147] purposed an advanced LT scheme applied to thin-film silicon-based solar cells, capable of actually overcoming such limits. They showed that optimized 3D optical modeling of thin-film hydrogenated nanocrystalline silicon (nc-Si:H) solar cells endowed with decoupled front and back textures, result in pronounced photocurrent densities (>36 mA/cm^2^), thus developing a suitable base for the fabrication of high-efficiency single and multi-junction thin-film solar cells. The simulated enhancements result from a gain in light absorption, especially in the NIR part of the spectrum close to the bandgap of nc-Si:H. Within this wavelength region, the material is weakly absorbing, whereas with the LT design of Isabella et al., significant absorption peaks are observed that can only be explained by the simultaneous excitation of guided resonances by front and rear textures. Using the same advanced LT employed for nc-Si:H, one would obtain a very high implied photocurrent density of 41.1 mA/cm^2^ for a device with a 2-μm thick absorber.

A recent breakthrough contribution by Li et al. [151] showed that the Green light absorption limit can also be approached without the need for complex LT geometries. With simple (yet smartly designed) grating structures, composed of checkerboard and/or penta arrangements, the photocurrent of thin-film silicon cells can be realistically doubled, thus revealing performance improvements at the level of the most sophisticated LT grating structures but here attained with much simpler (hence industrial-friendly) geometries.

#### 4.1.2. Thin-Film Solar Cells Improved with Front-Located Photonic Structures

Guided by the modeling studies, in the past few years, LT mechanisms have been successfully implemented in both rigid and flexible solar cell devices with promising experimental results. In this section, we summarize some of these results and the progress that has been achieved in practice. The LT mechanisms, in addition to allowing remarkable enhancement of the optical density of ultrathin PV films, can be fabricated with low-cost materials and integrated by industrial-scale procedures via inexpensive and large-area soft-lithography processes. Accordingly, colloidal lithography has been applied to pattern thin-film solar cells on a photonic length scale with low manufacturing costs.

The simplest CL-related LT implementation consists of using the close-packed array of colloidal spheres (Figure 2a), self-assembled on the front contact of the solar cells, not as a mask but as the photonic front structure itself. This allows forming the photonic structures with a single step performed as a post-process on the cells, as it avoids the additional colloidal masking steps. Such an approach was tested by Grandidier et al. [26], employing a monolayer of silica nanospheres deposited by Langmuir-Blodgett on the front TCO of hydrogenated amorphous silicon (a-Si:H) solar cells. In this design the dielectric colloids act as resonant Mie scatterers, coupling light into the absorber via their near-field proximity. At the same time, it acts in the far-field as a graded-index antireflection coating to further improve the photocurrent. Overall, even though the simple approach of Grandidier et al. allowed significant enhancements in the photocurrent (average of 6.3%) and efficiency (1.8%, reaching the absolute value of 11.1%), the attained gains are relatively low in comparison with those shown in Figure 10, predicted with optimized LT structures. One of the main reasons for this has to do with the low refractive index (*n*~1.5) of the silica spheres, while higher *n* values are desired for stronger antireflection and light scattering action, as indicated by the theoretical models [149].

**Figure 11:**
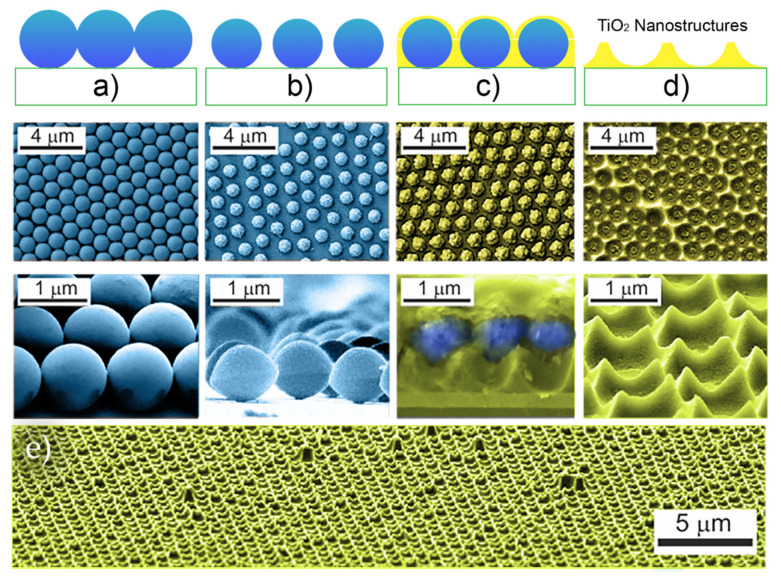
Schematic drawings and SEM pictures of the samples obtained after the different steps of the TiO_2_ nanostructure construction via CL on glass substrates: (**a**) a hexagonal array of colloidal PS spheres is patterned on the cell front, (**b**) O_2_ dry etching, (**c**) TiO_2_ is deposited, filling the inter-particle spaces, (**d**) the spheres are removed leaving an array of semi-spheroidal void-like features. The final TiO_2_ structure (**e**) uniformly covers the entire sample area. Adapted with permission from [33]. Copyright 2021 The Royal Society of Chemistry.

As such, photonic structures composed of high-index dielectric materials, (e.g., TiO_2_) as suggested in the simulation works reported in Section 4.1.1, should be capable of yielding much more pronounced enhancements. Given that, Sanchez-Sobrado et al. [33] (Figure 11) developed a CL technique aimed at engineering the TiO_2_ semi-spheroidal void-like array geometry of Figure 6b. The TiO_2_-based wave-optical structures were first integrated by CL on the front surface of a-Si:H thin-film absorbers, to optimize the parameters of the fabrication method while looking at the absorption enhancement caused in the films.

The method developed by the authors employs the four main steps illustrated in Figure 11: (a) deposition of periodic close-packed arrays of PS colloids (original diameter of 1.0 µm, 1.5 µm or 2.0 µm) which act as the mask, (b) shaping the particles and increasing their spacing via dry etching, (c) infiltration of TiO_2_ in the inter-particles spacing and (d) removal of the PS particles to leave only the nanostructured TiO_2_ layer [33].

It was demonstrated that when directly deposited on a-Si:H absorber films, such LT microstructures provided pronounced broadband absorption enhancement (27.3% on spectral average) in the a-Si:H medium relative to the unpatterned sample.

**Figure 12:**
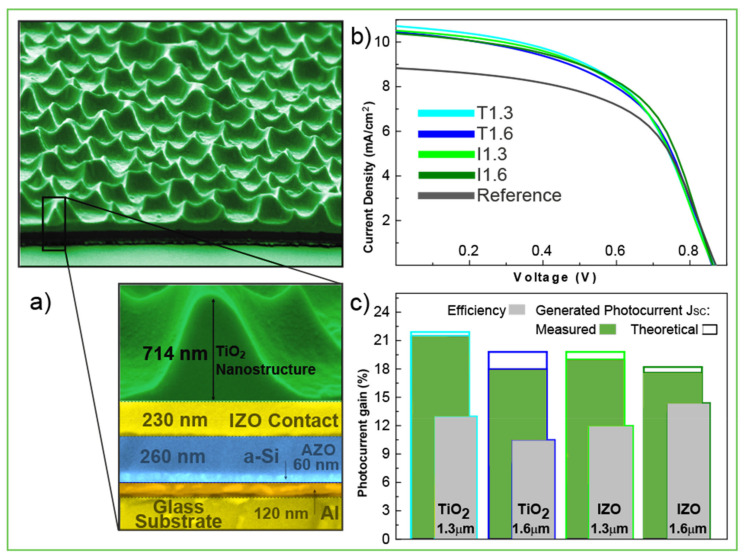
(**a**) SEM images of the cross-section of an a-Si:H solar cell coated with a TiO_2_-based LT structure (patterned by CL) over its front flat IZO contact; (**b**) Measured J-V curves of solar cells incorporating four different photonic coatings made of either TiO_2_ or IZO and formed with either 1.3 or 1.6 µm diameter PS spheres, and compared with the curve of the planar reference cell with no LT structure over the IZO contact; (**c**) measured and theoretically modeled enhancement, relative to the reference, of the generated current density (dark grey and empty bars, respectively), and the measured conversion efficiency (light grey bars) of the devices in (**b**). Adapted with permission from [48]. Copyright 2021 The Royal Society of Chemistry.

In subsequent work [48], the authors advanced to integrating this type of photonic structure in actual solar cell devices by CL. These were implemented as a top coating (see Figure 12), with two different materials tested for the photonic coatings in this work: TiO_2_, due to its high refraction index, and indium zinc oxide (IZO), for better optical and electrical coupling with the front contact of the cells composed of a flat IZO layer.

It was observed that all the different photonic structures applied on the a:Si:H cells produced significant broadband absorption enhancement, leading to systematic increases in the current (17.6–21.5%, Figure 12c), relative to that of the planar reference cells without the LT features. The TiO_2_ structures achieved higher optical performance than the IZO ones, as expected, mostly due to the higher refractive index and lower optical absorption of TiO_2_. Nevertheless, the extra electrical benefits (reduced sheet resistance) on the front electrode caused by the IZO structures allowed for the highest efficiency enhancement (Figure 12c) [48].

**Figure 13:**
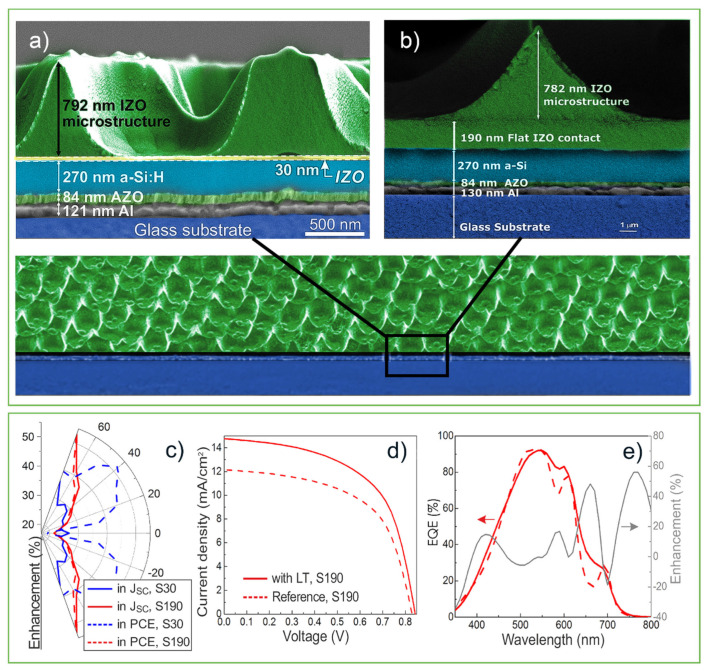
SEM cross-sections of a-Si:H solar cells with two different thicknesses of the flat IZO layer (30 (**a**) and 190 nm (**b**), respectively) located between the a-Si: H absorber and the front LT structures; (**c**) Polar plot representing the angular response of the solar cells with 30 nm, S30 (blue lines), and with 190 nm of IZO thickness, S190 (red lines), in terms of the gains attained in the short-circuit current density, (J_SC_, solid lines) and power-conversion efficiency (PCE, dashed lines) of the cells; (**d**) J-V curves and (**e**) external quantum efficiency obtained for S190 (190 nm of IZO thickness). Adapted with permission from [29]. Copyright 2021 Elsevier.

Given the promising results attained with the photonic-structured IZO front contacts, in a subsequent work Sanchez-Sobrado et al., (2020) [29] further improved the device architecture and CL process parameters to further optimize the geometry of the IZO structures and, importantly, their location concerning the Si absorber. This was achieved by optimization of the thickness (investigating from 30 to 250 nm) of the flat IZO layer beneath the LT structures, to allow an electrically effective front contact ideally coupled with the geometry of the CL-patterned IZO structures (Figure 13).

Here, the CL fabrication method consisted of first dispersing a colloidal suspension of 1.6 μm PS spheres in a water-ethanol mixture (1:3) at a solution concentration of 2.5% wt. Using the Langmuir–Blodgett technique [152], a close-packed monolayer was then deposited onto the flat IZO-coated surface of the a-Si:H cells.

The final IZO structure is revealed after removing the PS particle mask by a combination of Ar/CF_4_ reactive ion etching and a toluene bath.

As previously mentioned, the main parameter optimized in this work was the thickness of the flat IZO layer deposited on the cell before the CL process of integrating the top microstructured IZO. Such thickness defines the separation between the a-Si:H absorber and the photonic features, and the best results were attained with 30 nm and 190 nm thicknesses, shown in Figure 13a. These results reveal that the LT structures lead to a remarkable broadband enhancement of the total light absorbed by the devices, therefore leading to photocurrent gains (relative to reference planar cells) up to 26.7% with the 30 nm flat IZO space—Figure 13c. However, the best efficiency enhancement (23.1%) was attained with the optimized thickness of 190 nm for this layer (Figure 13d,e), as it provides the most favorable combination of optical and electrical gains.

Another important finding of this work was the remarkable LT gains attained at oblique light incidence. The angular response of the devices was evaluated by measuring the JV curve for a range of light incidence angles from 0° to 90°. The highest gain reaches 53.2% enhancement in photocurrent at 70° incidence angle for the cell with the optimized 190 nm thick flat IZO, and 52.2 in efficiency (at 40° incidence) with the ultrathin (30 nm) flat IZO (Figure 13e) [29].

#### 4.1.3. Photonic-Enhanced Perovskite Solar Cells and CL Compatibility

Perovskite-based PV materials, as thin-film absorbers for perovskite solar cells (PSCs) [153], have received unprecedented attention in both academia and industry due to the exceptionally rapid efficiency advancement from ~3.8% to >25.2% over the last decade [30,154]. As usual, for most PV technologies in the early stages of development, the progress was mainly accomplished by exploring the composition of perovskite-based materials and optimizing the fabrication process of the perovskite layer, as well as the quality of the cells’ interfaces to ensure efficient charge collection and to suppress unwanted recombination routes [155]. As such, PSCs have reached a stable point in the development phase, and optical strategies are now paramount to advance beyond its current record efficiency, particularly for the thinner perovskite layers which are attractive to enable device flexibility [156].

The rapid progress in this PV technology has enabled improved PSC deposition methods allowing conformal coating of the cell layers onto microstructured substrates, resulting in improved efficiencies. This is a promising path that has only recently started being unraveled for nano/micro-structuring in the PSCs field. Photonic microstructures can improve the cells’ absorption beyond the standard values achieved with planar devices, facilitating thickness reduction without compromising the output current and without degrading the electrical performance. On the other hand, the operational stability of the devices can also be improved by blocking the harmful higher-energy photons of UV radiation. This is particularly advantageous to assist in the stability of this less-matured PV technology which quickly degrades with UV exposure [52].

Recently, several LT schemes have been shown to improve the performance of PSCs, such as disordered micro-pyramids [155,156], nanojets, corrugated substrates, self-cleaning nanostructures, and micro-cones [157], as well as other approaches such as plasmonic nanoparticles, surface plasmon resonances, down and/or upconversion, etc. [158,159,160]. It is also observed that simple grating structures in the front [161] and back electrodes [162] enable enhancement in the light absorption and PSCs stability.

CL can play an incredible role in thin-film PV process technology due to its compatibility with large-scale (even roll-to-roll) manufacturing. The developed low-cost CL processes commented in the previous sections, for the integration of LT structures in silicon-based solar cells [29,31], can be adapted for photonic-structuring in PSCs. Previous theoretical contributions presented novel LT designs [27,28], operating in the wave-optics regime that demonstrated record photocurrent gains in PSCs via the incorporation of wavelength-sized features in the front electron transport layer (ETL) of the cells with a substrate configuration, similarly to the integration discussed in detail in Section 4.1.2. Interestingly, it was shown that the proposed front-patterned TiO_2_ LT coatings also lead to UV stability improvements [27], with even better results being achieved by incorporating another front-located luminescent down-shifting layer that can convert the harmful UV photons to non-harmful visible photons [28]. These structures can be straightforwardly fabricated using the same procedures of CL (Figure 6) as those developed for silicon-based solar cells [29,31], as further detailed next.

**Figure 14:**
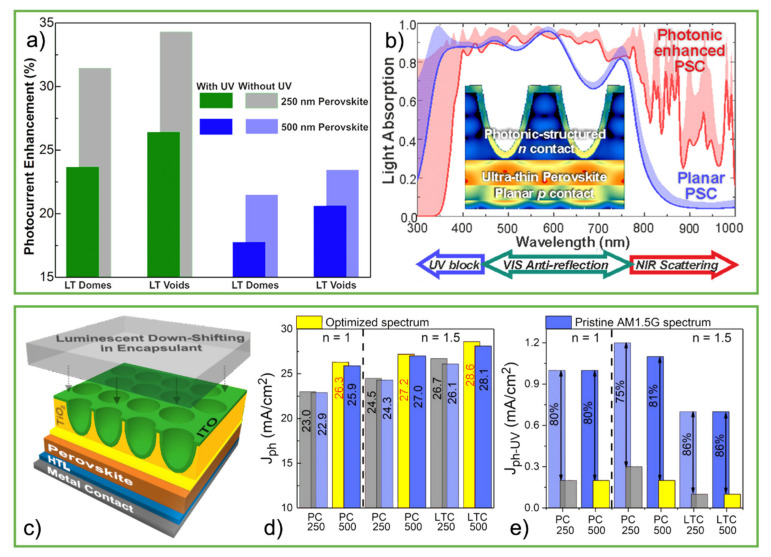
(**a**) Photocurrent enhancements attained with the optimized photonic structures presented in Haque’s work. (**b**) Light absorption spectra of PSCs with (photonic-enhanced) and without (planar) the optimized LT structures integrated into the ETL of the cell—a low absorption in the UV region indicates the desired blocking effect caused by the photonic structure. Adapted with permission from [27]. Copyright 2021 Elsevier. (**c**) Sketch of the luminescent down-shifting material encapsulating the photonic-structured PSC. (**d**) J_ph_ values obtained considering the full UV-Visible-NIR wavelength range (300–1000 nm), for two different refractive indexes (n) values of the encapsulating media. (**e**) UV photocurrent (J_ph-UV_) values for wavelengths ranging from 300 to 400 nm (the more transparent bars refer to the devices with 250 nm perovskite thickness, while the others refer to those with 500 nm). Adapted with permission from [28]. Copyright 2021 American Chemical Society.

The initial simulation work by Haque et al., (2019) [27] considered an inverted (substrate-type) PSC architecture, allowing the integration of the LT structures as a post-process on the front contact of the cells, in a similar way as in Figure 12 for thin-film silicon cells. The wave-optical structures optimized by the authors (Figure 14) for the inverted PSCs are also based on high-index dielectric (TiO_2_) micro-scale features, with semi-spheroidal geometries.

The optically lossless TiO_2_ material allows the structures to be patterned in the final processing steps, integrated into the top *n* contact of the cell. This, in turn, avoids structuring the cell layers, thence avoiding increased roughness and consequent electrical losses due to higher recombination. The electromagnetic field distribution simulations in the PSC structures were also carried out using the same FDTD method of Figure 10, and the main results are summarized in Figure 14a. In particular, the optimized array of TiO_2_ voids, which was shown to be optically favorable when compared with the domes, enables a photocurrent enhancement of 21% and 27% in PSCs with conventional (500 nm thick) and ultrathin (250 nm) perovskite layers, respectively.

The photocurrent enhancements attained with the optimized LT designs are mainly due to absorption improvements for wavelengths above 600 nm, which the authors attribute to strong antireflection (of visible light) and light scattering effects (of near-infrared), as shown in Figure 14b.

Furthermore, the TiO_2_ material of the structures advantageously acts as a UV blocking layer, as also shown in Figure 14b, protecting the perovskite from known degradation mechanisms caused by UV penetration [163,164,165]. Here the UV light is absorbed in the front TiO_2_ features and does not reach the perovskite, while the light at the longer visible plus NIR wavelengths is coupled and trapped within the cells, generating an overall enhanced photocurrent. UV blocking mechanisms as this one can enhance the operational stability of PSCs upon solar exposure, but inevitably they prevent the conversion of the UV energy by the cell, so they are not the ultimate solution when aiming for maximizing efficiency.

Alexandre et al. [28] presented an interesting approach to exploit such, otherwise lost, UV energy via the combined effects of LT and luminescent down-shifting (LDS). Simply put, this latter effect shifts higher energy light into lower-energy light. Considering the extensively studied degradation problems of PSCs with UV radiation [166,167], this method can bypass these unwanted mechanisms while also recurring to the energy coming from the UV photons.

The use of optimized LDS materials in the front encapsulation of the previous photonic-enhanced PSCs designed by Haque et al. (Figure 14c) led to an increase in photocurrent of at best 2% (~0.6 mA/cm^2^), which is almost half of the theoretical maximum current (1.4 mA/cm^2^) that could be gained from all the UV range. The optimum spectral down-shift was found to be from a central 350 nm UV wavelength to around 500 nm visible wavelength, matching well with the electrical performance peak of PSCs, which could imply that an increased device’s efficiency surpassed the projected gains. The LT cells also revealed a decrease in the harmful TiO_2_ photogeneration near the perovskite/TiO_2_ interface due to the LT structures’ UV shading effect. By assessing the UV penetration in the perovskite material (given by the UV generated photocurrent by the PSC) for the different simulated cells, reductions up to 86% (Figure 14d) were obtained when comparing photocurrent values for the original (pristine) and optimized down-shifted spectrum. Therefore, these analyses show that the use of LDS provides a more effective way to eliminate the unwanted effects of UV radiation in the perovskite, demonstrated by the hefty decrease in UV absorption coupled with the diminished TiO_2_ photoactivity from lower photogeneration, while also enabling additional power generation from the UV portion of the sunlight [28].

Although promising and compatible with CL structuring, the aforementioned LT implementations in Figure 14 require the photonic elements to be patterned on top of the planar cell layers during the final processing stages, which brings the risk of degrading the highly sensitive PSC materials located underneath [52]. Specifically, the fast degradation of PSCs with humidity exposure may render this class of devices incompatible with immersion or coating with aqueous solutions, as required in the first steps of the CL process (see Section 2.2).

**Figure 15:**
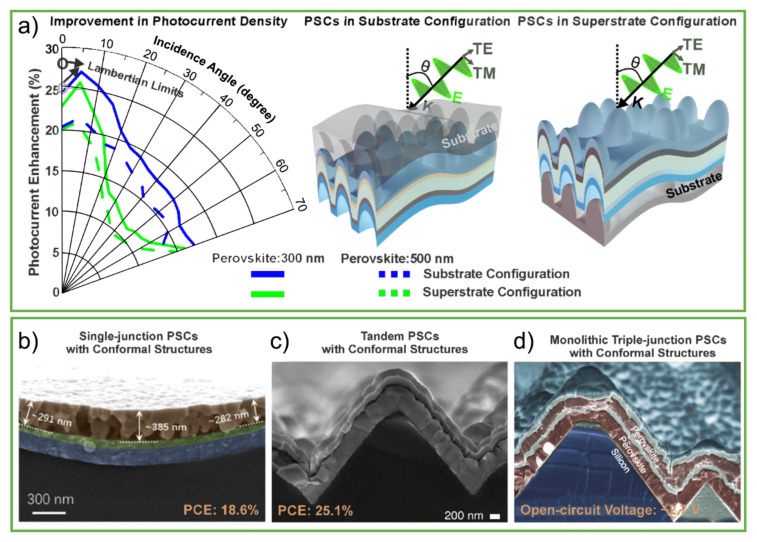
(**a**) The polar plot shows the photocurrent density gain as a function of the incidence angle (θ), attained with the optimized photonic-structured PSCs in a conformal architecture, for both the substrate and superstrate cell configurations illustrated in the middle and right sketches. The Lambertian limits of LT in PSCs, in the geometrical optics regime, are also indicated for normal incidence angle (θ = 0°) in the left plot. Adapted with permission from [30]. Copyright 2021 Elsevier. (**b**) SEM image of a single-junction PSC conformally coated onto a textured glass substrate. Adapted with permission from [168]. Copyright 2021 Elsevier. (**c**) SEM image of a tandem perovskite/silicon cell with ~25.1% efficiency, in which the PSC is conformally coated onto the textured silicon wafer-based bottom cell [169]. (**d**) SEM image of a triple-junction perovskite/perovskite/silicon cell in which the top and middle PSCs are also conformally deposited onto textured silicon. Adapted with permission from [170]. Copyright 2021 American Chemical Society.

Given this, Haque et al., (2020) [30] investigated a more industrially viable LT strategy consisting of the development of photonic substrates used for subsequent PSC deposition. The authors have shown that it is possible to achieve pronounced broadband absorption enhancement provided by the LT-patterned substrates, relative to planar cells, which was also observed for a broad range of incidence angles (0–70 degrees), as seen in Figure 15a. Apart from substantial light absorption enhancement, this approach has a significant advantage for its practical compatibility with PSC technology over the previous ones, as the PSC layers can be wet coated by traditional methods onto a substrate already patterned with the designed LT structure.

This makes the full photonic integration independent of the fabrication of the PSCs. Moreover, this is a potentially more cost-effective approach since it requires no extra materials (coatings) for the LT structuring.

However, the LT designs of Figure 15a are only achievable with highly conformal PSC deposition methods, capable of coating the cell layers onto the micro-patterned substrate surfaces without defect formation. In that respect, the work of Wang et al. [168] is an important contribution, as the authors developed a recrystallization treatment that enabled the conformal coating of high-quality perovskite layers onto textured glass with features in the order of the micrometer, resulting in 18.6% PSC efficiency with a ~300 nm thin perovskite absorber, as seen in Figure 15b.

Conformal deposition methods have also shown remarkable potential in monolithic tandem PV cells, in which perovskite-based top cells are coated onto fully textured crystalline silicon bottom cells. That is the case of the perovskite/silicon double-junctions developed by Aydin et al. [169] with 25.1% efficiency (Figure 15c) and the perovskite/perovskite/silicon triple-junctions (Figure 15d) of Werner et al. [170] reaching ~2.7 V.

Within this section, we went through some of the latest trends on LT strategies for PSCs, revealing promising modeling results with optimized designs for efficiency and stability improvement. We also discuss the first experimental steps that have been recently undertaken to circumvent the challenges associated with the practical realization of photonic-structured PSCs, which is a new avenue for CL implementation with enormous potential in the PV field.

### 4.2. Micro-Meshed Transparent Electrodes

Another highly promising solution offered by nano/micro-structuring is the development of high-performing transparent electrodes, with strong interest not only for the illuminated contacts of solar cells but also for many other optoelectronic applications. Nano/microstructured metallic films (termed micro-mesh electrodes, MMEs) offer an exciting alternative to flat TCO (transparent conductive oxide) layers, and CL was shown to be one of the most promising approaches to produce industrial-compatible MMEs with excellent properties [62].

State-of-the-art indium tin oxide (ITO) based TCOs are ubiquitously spread in optoelectronic technologies as they are in the illuminated contacts of solar cells, in displays, touch screens, and light-emitting diodes, with low sheet resistances between 8 and 12 Ω/□ (for commercial ITO) [171] and optical transmittance above 80% in the visible range [62,172]. However, as with most TCO materials, their free electrons cause strong parasitic absorption losses in the NIR range, limiting their performance for solar cell applications. This is further compounded by the rising cost of In, a rare material, while alternative TCOs made with Earth-abundant materials (e.g., based in ZnO [173]) offer reduced figures-of-merit in terms of transmittance-over-resistance ratio. Furthermore, ITO is usually deposited by costly DC-magnetron sputtering involving high temperatures (up to 300 °C) [174], making it unsuitable for deposition on thermal-sensitive materials. Moreover, ITO is brittle, making it also less attractive for flexible electronics as well as resistive touch screens [175].

**Figure 16:**
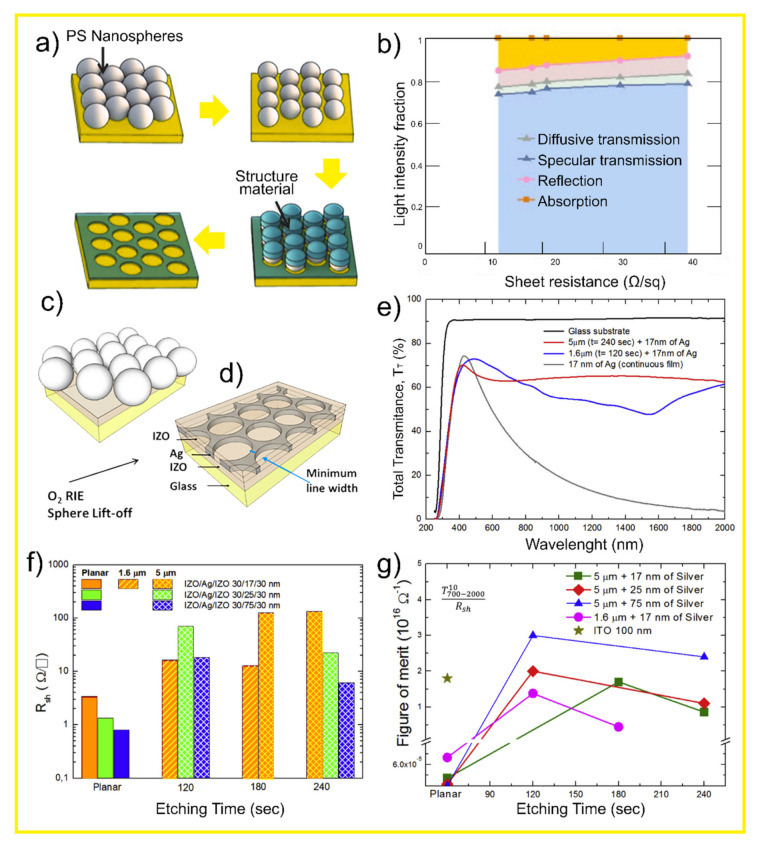
(**a**) Schematic of the Cu MME fabrication process by CL. (**b**) Plot of the measured diffuse transmission, specular transmission, reflection, and absorption at λ = 550 nm versus sheet resistance for a variety of Cu MMEs on quartz. Adapted with permission from [172]. Copyright 2021 American Chemical Society. (**c**,**d**) Schematic of the CL fabrication of Ag MMEs, showing the initially deposited PS sphere mask (**c**) and the final structure of the IZO/Ag grid/IZO electrode (**d**), in which the top and bottom IZO layers are ultrathin (30 nm). (**e**) Transmittance of samples with 17 nm thick Ag grids (blue and red lines), produced with distinct sphere size and etching time, compared to a continuous Ag film (grey line)—high transparency can be noted in the NIR region. (**f**) Sheet resistance of the electrodes fabricated with different diameters of PS spheres (1.6 or 5 μm), etching time, and Ag thickness. (**g**) Haacke’s figure-of-merit (expression in inset) for the main MMEs attained in this work, in comparison with a state-of-the-art TCO film made of ITO and with continuous (planar) Ag layers. Adapted with permission from [62]. Copyright 2021 Elsevier.

A solution to mitigate the issues of state-of-the-art TCOs is to use a metallic micro-grid (MMEs) sandwiched between ultrathin layers of TCO, which allows a pronounced increase in transparency (especially in the red-NIR region) while maintaining high sheet conductance; thus, offering an exciting alternative to flat TCOs. CL provides a promising approach to produce industrial-compatible MMEs, as demonstrated with the copper (Cu) nanomeshes developed by Gao et al., (2014) [175] exhibiting an excellent diffuse transmission of 80% and sheet resistance of 17 Ω/□ (Figure 16a,b).

More recently, Torrisi et al., (2019) [62] have shown the use of CL to create silver (Ag) micro-grids that were sandwiched between TCO layers. First, a PS colloidal microsphere monolayer (deposited using the Langmuir–Blodgett technique) is submitted to plasma etching to serve as a deposition mask. The subsequent evaporation of Ag throughout this mask, followed by lift-off of the colloids, creates a metallic grid as shown in Figure 16c,d. Compared to conventional transparent electrodes (i.e., ITO), excellent electrical and optical characteristics have been accomplished with such TCO/Ag micro-grid/TCO multilayers, such as sheet resistances below 10 Ω/□ and pronouncedly higher near-infrared transmittance, using different layer thicknesses and mesh dimensions (Figure 16e).

The structural parameters of the produced mesh (openings, line width, and thickness) play a vital role in the electrical and optical performance of the transparent electrodes. Larger hole structures, attained with large sizes of the PS colloids, result in excellent transmittance values at the cost of increased sheet resistance. Alternatively, meshes with smaller holes are less transparent but have a lower resistance, permitting higher currents (Figure 16f). To reduce the sheet resistance values, one can also increase the metal grid thickness, by evaporating more Ag material, without having a large effect on transparency [62]. Finally, the best performing correlation between optical and electrical properties (Figure 16g) is attained with 5 µm spheres and 17 nm Ag thickness, either (120 or 240s RIE).

It is also believed that the use of metallic grids for electrodes when compared to continuous layers, can bring several advantages for flexible transparent materials. In particular, honeycomb grids as produced via CL allow the highest flexural robustness with thinner MME structures [175], due to the fact that the honeycomb lattice enables the highest packing density in 2D arrays. Overall, the use of metallic micro-grids opens new and promising paths for transparent electronics, offering additional degrees of freedom for further electro-optical improvements. Specifically, their much higher transmission if the red-NIR region can be crucial for applications such as smart windows, low-energy photovoltaic devices, and as intermediate contacts in multi-terminal multi-junction (tandem) solar cell architectures [176,177].

### 4.3. Self-Cleaning with Photonic Structuring

Unavoidable environmental degradation is a major cause of efficiency losses in solar panels over time. Phenomena such as the formation of hot-spots (areas of large heat dissipation) caused by partial shading of solar cells can be responsible for large efficiency losses [74,143]. Among different solutions mentioned at the beginning of Section 4, the use of a self-cleaning coating appears to be the simplest and most fitting approach for large-scale PV installations [74], as no mechanical structure is required for its cleaning function.

Through superhydrophobic coatings, the concept of self-cleaning glass materials showed a maturing research trend between 2009 and 2017, with 1125 research articles being published (ScienceDirect keywords: “self-cleaning glass, superhydrophobic glass”). These coatings can be fabricated using top-down approaches such as template-based, photolithographic, and surface plasma treatment [178], or top-down methods such as chemical modification [179], colloidal assembly [180], layer-by-layer deposition [181], and sol-gel methods [182] can also be applied [74].

**Figure 17:**
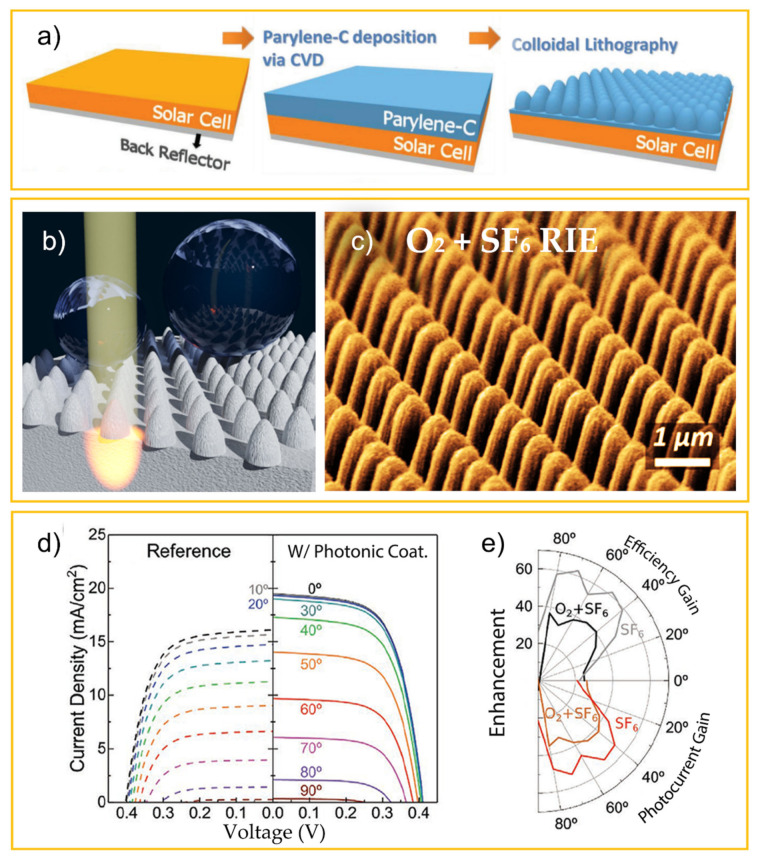
(**a**) Depiction of the patterning of the photonic-structured parylene-C coating on the transparent front contact of thin-film silicon solar cells, via chemical vapor deposition (CVD), followed by CL with a patterning approach similar to that of Figure 6b. (**b**) Artistic image of the photonic coating on the solar cell, illustrating its superhydrophobic surface where water droplets easily roll-off. The microstructured parylene-C provides LT while also acting as a water-repellent protective layer, which allows an effective self-cleaning functionality. (**c**) SEM image of the parylene-C surface microstructured via the CL process (O_2_ + SF_6_ RIE). (**d**) 1-Sun JV curves of the solar cells before (uncoated reference, left curves) and after (right curves) coating with the photonic-structured parylene-C shown in images (**c**,**d**), for illumination angles varying from 0° (normal to cell’s surface) to 90° (parallel to the surface). (**e**) Polar plot presenting the angular dependence of the gain in efficiency (top) and photocurrent (bottom) of the LT-enhanced solar cells relative to the planar references. Adapted with permission from [31]. Copyright 2021 John Wiley and Sons.

An important application for PV technology was presented by Centeno et al., (2020) [31], showing a simple, low-temperature, low-cost, and scalable CL method (Figure 17a) to engineer parylene-C (poly(chloro-p-xylylene)) coatings with encapsulating properties that endow effective light-trapping (LT) and water-repelling functionality when applied in thin-film solar cells (Figure 17b–e). Parylene-C was used as the preferred coating material, as it is a polymer with excellent barrier properties for encapsulation [183,184] and low water-adhesion surface energy.

It also benefits from optical transparency, adequate refractive index, outstanding flexibility, and mechanical strength. Moreover, parylene is deposited via chemical vapor deposition, which allows for the deposition of more uniform coatings at room temperature, thus making it usable with non-flat temperature-sensitive materials [183,185,186].

In this work, the hydrophobicity of parylene was controlled by simultaneously adjusting the surface corrugations (i.e., roughness, patterned features) and surface chemical composition with the CL patterning process following Figure 17a. In this way, a parylene-C film was micro-patterned with a hexagonal array of cones (see SEM in Figure 17c) after being coated on the front TCO of nc-Si:H solar cells [31].

These microstructured parylene coatings also enable outstanding antireflection and light scattering properties that were shown to enhance the solar cells’ photocurrent by up to 23.6% with normal incident light. Furthermore, the cells’ angular response was also improved, similarly to the previous work of Sanchez-Sobrado et al., (2020) [29] shown in Figure 13. Figure 17d displays the JV curves with an illumination angle ranging from 0° to 90°. The polar plot of Figure 17e shows the influence of the illumination angle on the efficiency and J_SC_ enhancement relative to the flat reference cell without the front microstructures.

These enhancement values increase with the angle until ~50°, with peak enhancements up to 52% and 61% in J_SC_ and efficiency, respectively, while both V_OC_ and FF (fill-factor) were only marginally reduced with increasing angle. Such gains for oblique incident light translate to an estimated average daily enhancement of ~35% in the generated energy, as compared with the daily energy delivered by the uncoated reference cells.

### 4.4. Overview of Achievements

The following tables list the best results concerning the CL-patterned PV devices (relative to the unpatterned references) commented along this section (see Figure 9 schematic). Table 1 summarizes the optimized computational designs and Table 2 the experimental outcomes.

## 5. Other Applications of Colloidal Lithography: Biological Cell Studies

Apart from the engineering of photonic solutions for optoelectronic-related technologies, such as photovoltaics, several nano/micro-structuring colloidal lithography (CL) approaches have been used to develop breakthrough advances in most various scientific areas. Among those, the biology community has been gaining increasing interest in CL, as overviewed next to show the bigger picture of promising applications.

Micro and nanofabrication techniques have revolutionized the pharmaceutical and medical fields. Complex geometries can be reproduced with technologies such as photolithography and particularly colloidal lithography [187]. The interaction of cells with material surfaces has been widely studied, having relevance for the function of medical devices, as well as to gain a better understanding of cellular action in vivo and in vitro [188]. In this field, nano-topography has shown that nanoscale features can strongly influence cell morphology, adhesion, proliferation, and gene regulation, but the mechanisms mediating this cellular response remain unclear [189]. Nano-topographies produced by CL may be of great importance when considering how cells respond to their environment, allowing for the elucidation of nano-feature effects on cell behavior. This appears to significantly alter the morphology of endothelial cells, epithelial cells, epitenon cells, macrophages, osteoblasts, and fibroblasts [190]. The cell reactions can be controlled by the surface characteristics morphology, chemistry, and viscoelastic properties. Investigations on the influence of nano-topographies in the cell response require surfaces patterned over large areas with high reproducibility, high throughput, and fabricated in biocompatible materials [189,191].

For instance, the ability of fibrinogen molecules to specifically bind to receptors in platelet membranes has been correlated with both nanoscale chemistry (hydrophobic and hydrophilic) and surface topography (nanopits and planar surface) [192]. Thus, CL was used to create a continuous thin-film of TiO_2_ with a distribution of nanopits with 40 nm diameter and 10 nm depth.

**Figure 18:**
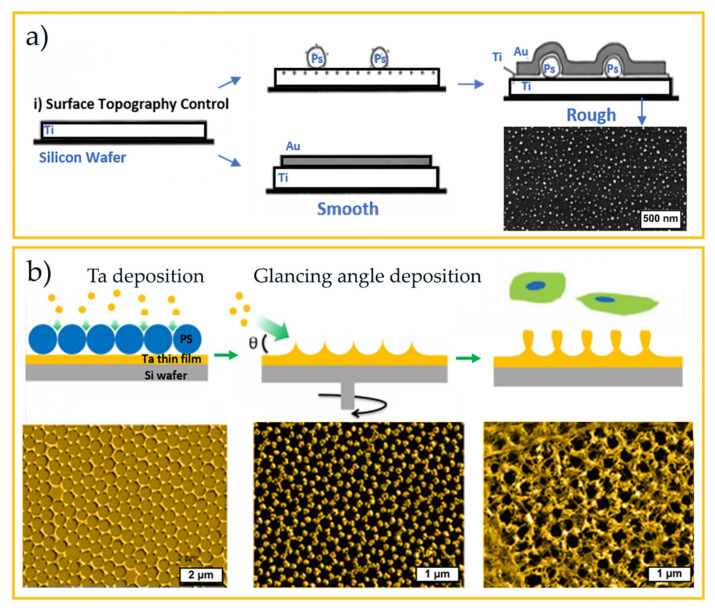
(**a**) Schematic illustration of prepared substrates with controlled nanotopography using CL and surface chemistry by alkanethiol self-assembly: i) smooth substrates were obtained by depositing titanium and gold layers onto silicon wafers; rough substrates were obtained by adhesion of negatively charged polystyrene colloidal particles to a smooth positively charged surface, followed by coating with a thin layer of gold. Adapted with permission from [193]. Copyright 2021 American Chemical Society. (**b**) Schematic structure of fabrication of ordered tantalum (Ta) nanotopographies using a combination of CL and glancing angle deposition techniques; and corresponding SEM images. Adapted with permission from [194]. Copyright 2021 American Chemical Society.

Similarly, collagen adsorption in terms of both adsorbed amount and the supramolecular organization was investigated concerning the influence of substrate surface topography (smooth surface versus nano-protrusions) and surface chemistry (CH_3_ versus OH groups). CL and functionalization with alkanethiol were used to create model substrates with these controlled topographical and chemical surface properties [193] (Figure 18a). The surface chemistry was found to control collagen protein adsorption, while chemistry and topography control collagen protein conformation.

The application of nanotechnology in the field of regenerative medicine has opened a new realm of advancement in this field. The human fibroblasts have a pivotal role during the initial phases of implant integration and resultant healing processes. Concerning the tissue–implant interface, in vitro investigations indicate that micro-topography can be used to control cell behavior, including that of fibroblasts, via initial adhesive interactions [191,195]. In this respect, CL was used to fabricate irregular nanotopographies with features of either 20 or 50 nm diameter pillars. These nano-pillar topographies modified fibroblast adhesion, morphology, and behavior relative to those deposited on planar substrates. The fibroblast adhesion is observed to be greater on substrates patterned with 20 and 50 nm diameter colloidal topographies, which result in increased fibroblast adhesion at 20 min and 60 min. Although the colloidal topographies alter the fibroblast adhesion, morphology, and behavior when compared to control conditions (planar substrates), for 180 min the adhesion is similar to planar surfaces [191].

Andersson et al. [188] investigated how pancreatic epithelial cell lines (AR4-2J) respond to surface structures (titanium pillars) of systematically increasing size. For this study, a surface with the same surface chemistry and similar surface roughness (but with increasing size of column diameters from 60 to 170 nm) was produced via CL. The changes in the epithelial cells were studied by evaluating cell area and cell shape. It was observed that the larger the feature, the more spread the cells became [188].

The behavior of primary human adipose-derived stem cells was studied on highly ordered bowl arrays (Figure 18b). PS colloids (722 nm diameter) were self-assembled into a hexagonally close-packed crystal array at the water-air interface, transferred onto a biocompatible Ta surface, and used as a mask to generate an ordered Ta pattern. The results showed that ordered Ta nanotopographies inhibited cell spreading, focal adhesion formation, and filopodia extension when the surface roughness and feature height increased [194]. It was also demonstrated that by changing the feature size, the ordered topographies could alter stem cell adhesion and differentiation.

These remarkable investigations have been expanding our knowledge in cell-surface interactions, which is of great interest in biomaterials, tissue engineering, and cell therapy applications; being CL a preferential industry-compatible technique for the needed surface structuring.

## 6. Conclusions

As nano/microstructures fabrication grows in importance in a wide range of areas from electronics through optics, microanalysis, combinatorial synthesis, displays, cell biology, etc., the research on ever-more cost-effective patterning methods will undoubtedly continue to increase.

In the past decade, colloidal lithography has grown to become one of the most attractive soft-patterning techniques, and a solid alternative to conventional hard-patterning processes. Its main characteristic is that it embodies a simple and inexpensive way of producing the lithographic mask, using monodisperse colloidal particles self-assembled on the target surface. This method offers advantages in applications in which photolithography falters, for instance, manufacturing below the (sub-wavelength) scale of 100 nm, patterning on non-planar surfaces, fabrication of three-dimensional structures, patterning functional materials other than photoresists, among other types of surface modifications. Another key advantage is that colloidal lithography allows the formation of nano/micro-scale structures of virtually any material, via scalable process steps that are compatible with industrial mass-production requirements. As such, it is highly attractive for crafting photonic schemes, such as those discussed here for photovoltaic applications, since it enables the precise engineering of long-range ordered wavelength-sized features, with the materials and geometries appropriate for efficient light-trapping.

This article overviewed some of the most promising nano/micro-structuring solutions in the growing field of photovoltaics, where colloidal lithography was shown to be a preferential patterning technique for the practical realization of the concepts in industrially attractive ways. In particular, photonic-enhanced photovoltaics has been shown to circumvent many of the conventional shortcomings of current solar cell technologies, such as the reflection losses and impaired light absorption especially in thin-film devices; as well as enabling other interesting functionalities such as improved transparent contacts and self-cleaning due to the high aspect ratio of the photonic microstructures.

## Figures and Tables

**Table 1:** Overview of simulation results on photonic microstructures for thin-film PV.

Authors	Year [Ref.]	Simulated Structure	Best Results
Mendes et al.	2016 [149]	Front-located TiO_2_ domes on planar Si solar cells	Photocurrent gain of 50% for thin c-Si cells with 1.5 µm thick absorber
	2018 [142]	Front-located TiO_2_/TCO wavelength-sized dome/void arrays	Photocurrent gains of 37%, 27%, and 48% in 100 nm a-Si, 300 nm a-Si, and 1.5 µm c-Si, respectively
Isabella et al.	2018 [148]	Thin-film nc-Si:H solar cells with decoupled front and back textures	Photocurrent densities reaching 41.1 mA/cm2 with 2-μm thick nc-Si:H absorber.
Haque et al.	2019 [27]	TiO_2_ LT structures integrated on the front contact of flat Perovskite solar cells (PSC)	Photocurrent gains up to 27% with planar ultrathin (250 nm) perovskite layers, together with UV blocking effect granting improved stability.
	2020 [30]	Microstructured PSCs on transparent and opaque photonic substrates	Photocurrent gains of 22.8% and 24.4%, respectively with transparent and opaque substrates, with 300 nm perovskite.
Alexandre et al.	2019 [28]	Combined luminescent down-shifting and front-located photonic structures on PSCs	Photocurrent gains similar to Haque et al., together with up to 86% reduction of UV penetration in the perovskite layer.

**Table 2:** Overview of experimental results on CL-fabricated microstructures for thin-film PV.

Authors	Year [Ref.]	Experimental Structure	Best Results
Grandidier et al.	2013 [26]	Close-packed monolayer of silica microspheres on the front of a-Si:H solar cells	Spheres acting as resonant Mie scatterers provide photocurrent and efficiency gains up to 8.9% and 11.1%, respectively.
Sanchez-Sobrado et al.	2017 [33]	Front-located TiO_2_ microstructures on a-Si:H thin-film absorbers	Absorption enhancement of 27.3% on spectral average in 300 nm a-Si:H absorbers
	2019 [48]	TiO_2_ or IZO structures integrated into front contact of a-Si:H solar cells	Photocurrent gains up to 21.5% with TiO_2_ (better optically) and efficiency gain up to 14.4% with IZO (better electrically) LT structures.
	2020 [29]	Front-located IZO structures on a-Si:H solar cells with optimized parameters	Photocurrent and efficiency gains up to 26.7% and 23.1%, respectively, while gains >50% are attained at an oblique incidence.
Centeno et al.	2020 [31]	Micro-patterned parylene-C film with micro-cone array coated on the front TCO of nc-Si:H solar cells.	Water repellant (self-cleaning) surface, granting photocurrent and efficiency gains up to 23.6%, and up to ~60% at oblique incidence.
Gao et al.	2014 [172]	Cu micro-mesh electrode as a transparent conductive material	Diffuse transmission of 80% in the visible range; sheet resistance of 17 Ω/□ on quartz; high flexural robustness.
Torrisi et al.	2019 [62]	Ag micro-grids sandwiched between ultrathin TCO layers	Sheet resistances below 10 Ω/□ and near-infrared transmittance over 50%.

## Data Availability

Not applicable.
